# PYHIN Proteins and HPV: Role in the Pathogenesis of Head and Neck Squamous Cell Carcinoma

**DOI:** 10.3390/microorganisms8010014

**Published:** 2019-12-20

**Authors:** Giuseppe Riva, Matteo Biolatti, Giancarlo Pecorari, Valentina Dell’Oste, Santo Landolfo

**Affiliations:** 1 Otorhinolaryngology Division, Department of Surgical Sciences, University of Turin, 10126 Turin, Italy; giuseppe.riva@unito.it (G.R.); giancarlo.pecorari@unito.it (G.P.); 2 Laboratory of Pathogenesis of Viral Infections, Department of Public Health and Pediatrics, School of Medicine, University of Turin, 10126 Turin, Italy; matteo.biolatti@unito.it (M.B.); valentina.delloste@unito.it (V.D.)

**Keywords:** HPV, head and neck cancer, PYHIN proteins, IFI16, AIM2

## Abstract

In the last decades, the human papillomavirus (HPV) emerged as an etiological cause of head and neck squamous cell carcinoma (HNSCC), especially in the oropharynx. The role of two intracellular DNA sensors, which belong to the PYHIN family (interferon-inducible protein 16 (IFI16) and absent in melanoma 2 protein (AIM2)), has been analyzed in relation to HPV infection and head and neck carcinogenesis. In particular, IFI16 and AIM2 expression depends on HPV infection in HNSCC. They represent viral restriction factors and are key components of the intrinsic immunity activated against different viruses, including HPV. This review analyzed and summarized the recent findings about the role of PYHIN proteins in HPV^+^ and HPV^−^ HNSCC.

## 1. Introduction

Head and neck squamous cell carcinoma (HNSCC) represents the sixth most prevalent type of cancer, including about 6% of all cases of tumors worldwide. In the male and female population, its incidence is of 15.2 and 4.6/100,000 people, respectively, accounting for approximately 650,000 new cases and 350,000 deaths per year [1].

Although the two major risk factors for HNSCC are alcohol and tobacco abuse, which act synergistically, the human papillomavirus (HPV) has recently emerged as one important independent risk factor for the development of these tumors, especially at the level of the oropharynx, with the tonsils and the base of the tongue being the most vulnerable sites. In particular, HPV infection has been directly associated with increased incidence of oropharyngeal tumors in men younger than 50 years without a prior history of tobacco and alcohol consumption [2]. HPV infection is also associated with a different mutational profile and better survival [3,4].

The PYRIN and HIN domain(PYHIN) family consists of five interferon-inducible proteins that have recently emerged as intracellular DNA sensors. These include the interferon-inducible protein 16 (IFI16), absent in melanoma 2 protein (AIM2), myeloid cell nuclear differentiation antigen (MNDA), pyrin and HIN domain family 1 (PYHIN1 or IFIX), and PYRIN domain (PYD)-only protein 3 (POP3) [5,6]. The gene cluster is positioned on the human chromosome 1 position (23) long (q) arm. Each PYHIN protein contains an N-terminal PYRIN domain (PYD), with some of them harboring one or more C-terminal DNA binding domains (HIN), classified as HIN A, B, and C type [7]. Variable family expansion of these interferon-inducible proteins has been detected in other mammals, with at least thirteen family members present in mice (interferon-inducible (Ifi) 200 cluster) proteins, which contain at least one copy of a conserved 200 amino acid domain, responsible for common functions [5,6].

In this review, we will describe our current understanding of the emerging role of PYHIN proteins in HNSCC pathogenesis, focusing in particular on IFI16 and AIM2, whose expression depends on HPV infection [8,9,10,11,12,13,14].

## 2. Head and Neck Squamous Cell Carcinoma

HNSCC arises from mucosal epithelial cells of the upper airways (i.e., oral cavity, oropharynx, hypopharynx, and larynx). Its complex anatomical origin, as well as its different etiologies and molecular alterations, makes this tumor intrinsically heterogeneous [2,3].

Although alcohol and smoking abuse represent classical risk factors for HNSCC, infection by high-risk HPV serotypes has increased the prevalence of this tumor, mainly in the oropharyngeal area [15]. Indeed, about 90% of HPV-related oropharyngeal tumors are due to the high-risk HPV serotype 16 [16]. Thus, HNSCCs can be grouped into HPV-positive (HPV^+^) and HPV-negative (HPV^−^) tumors, each with distinct clinical presentation, molecular profiles, and prognosis [17,18,19,20]. In particular, HPV^+^ HNSCCs show a better response to chemoradiotherapy and survival [21].

HPV^+^ HNSCC more often affects young non-smoking and non-drinking individuals compared to HPV^−^ HNSCC. Moreover, HPV^+^ HNSCC generally presents itself as a small primary tumor with large nodal metastases [21]. Due to the different clinical presentation and the more favorable outcome of HPV^+^ vs. HPV^−^ oropharyngeal squamous cell carcinoma, the tumor-node-metastasis (TNM) staging for oropharyngeal tumors has been recently changed by the American Joint Committee on Cancer (AJCC) and Union for International Cancer Control (UICC) to better represent this emerging feature [22].

HPV^+^ tumors comprise approximately 22%–47% of all oropharyngeal HNSCCs [15], with the highest prevalence being in South America (53%) and central, eastern, and northern Europe (50%) and the lowest in southern Europe (9%) [15]. The fraction of HPV-linked oropharyngeal HNSCC increased from 7.2%, in 1990–1994, to 32.7%, in 2010–2012, probably due to changes in sexual behavior as HPV^+^ HNSCC are positively associated with oral sex [23].

HPV infection in non-oropharyngeal HNSCC is less frequent. A systematic review reported an HPV DNA prevalence for oral and laryngeal tumors of 23.5% and 24.0%, respectively [16]. However, another meta-analysis showed that a large number of these HPV^+^ HNSCCs were negative for *E6* and *E7* gene expression [24]. Although mRNA expression of these two viral oncogenes is considered to be the most reliable biomarker of HPV-mediated transformation, a recent study has only detected HPV DNA and *E6/E7* mRNA in 3.9% and 3.1% of oral and laryngeal tumors, respectively [15].

In the last few decades, a number of molecular changes mainly involving oncogenes and tumor suppressor genes (e.g., *NOTCH1*, *p16*, and *TP53*) or genes regulating the cell cycle (e.g., *EGFR* and *cyclin D1*) have been identified in HNSCC, with *NOTCH1* and *TP53* being the most frequently found mutated genes [4]. Importantly, different expression profiles have been reported concerning HPV^−^ and HPV^+^ HNSCC [3]. In this regard, HPV E6 and E7 have been shown to contribute to tumor development through inactivation of p53 and retinoblastoma protein (pRb) [24,25,26]. The loss of pRb increases p16 protein expression, which in turn inhibits cyclin D1/cyclin-dependent kinase 4/6 (CDK4/6) signaling required for G_1_/S transition. Consequently, p16 is generally regarded as a bona fide molecular marker of HPV infection, which has recently led to modification of the TNM staging to include p16 positivity as a surrogate for HPV status [22]. However, the fact that p16 overexpression is not always associated with HPV DNA positivity in HNSCC implies that this protein might not be a reliable screening marker of HPV infection in this tumor type [27,28].

HPV^−^ and HPV^+^ HNSCCs show different molecular profiles (Table 1). The most common genetic alterations in HPV^−^ HNSCC are the losses of chromosomes 3p and 9p as well as *TP53* mutations [29]. The loss of the tumor suppressor gene *CDKN2A*, which encodes for p16INK4A, along with the amplification of *cyclin D1* drives cells through the G_1_/S checkpoint and contributes to DNA replication [30]. Typically, dysregulated DNA replication leads to DNA damage and p53 activation, which results in cell cycle arrest and apoptosis. However, this does not generally occur in HPV^−^ HNSCC cells where *TP53* is often inactivated by missense mutations and allelic loss [29]. Indeed, TP53 somatic mutations are typically found in 30%–75% of HNSCCs and correlate with poor survival in invasive carcinomas [31,32,33].

*NOTCH1*, known to regulate normal cell differentiation, lineage commitment, and embryonic development, is the second most frequently mutated gene in HNSCC (14%–15% of cases) [17]. The prevalence of loss-of-function mutations of *NOTCH1* in HNSCC has led to the hypothesis that this protein acts as a tumor suppressor rather than as an oncogene in this type of tumor [34]. Other genes involved in HPV^−^ HNSCC include the epidermal growth factor receptor (*EGFR*), often amplified in HNSCCs and related to cell cycle control [35], and *Met*, a receptor tyrosine kinase often associated with enhanced migration, invasion, and angiogenesis when overexpressed in cancer [36].

On the other hand, a number of epigenetic and genomic studies have shown how the majority of commonly altered genes in HPV^−^ HNSCCs are often unaltered in their HPV^+^ counterparts [20]. This is particularly true for the tumor suppressor gene *TP53* as HPV^+^ HNSCCs express this gene in its wild-type conformation, whereas HPV^−^ HNSCCs generally harbor a mutated form [20]. Moreover, HPV^+^ HNSCCs have a lower average number of mutations per tumor, and rarely display p16INK4A loss-of-function compared with HPV^−^ HNSCCs [17,34,37,38].

Another interesting difference concerns *PIK3CA* mutation hotspots. *PIK3CA* encodes p110α, a catalytic subunit of phosphoinositide 3-kinase (PI3K), which activates the v-akt murine thymoma viral oncogene(AKT) signaling pathway. HPV^+^ HNSCCs carry mutations in the helical domain of this protein, whereas HPV^−^ tumors harbor mutations throughout the entire gene, albeit those in the helical and kinase domains are more frequently observed. In particular, *PIK3CA* amplification and/or mutation can be found in 34% of HPV^−^ and 56% of HPV^+^ HNSCCs [17].

More recently, our group has shown that the apolipoprotein B mRNA-editing enzyme catalytic subunit (APOBEC) family of cytidine deaminases plays a role in HPV^+^ HNSCC [14], in line with the notion that an APOBEC-induced mutational signature can determine a specific mutational profile in HPV^+^ tumors [39]. APOBEC induction is not only triggered by virus infection but may also be a result of gene amplification [40]. Interestingly, HPV^−^ HNSCC presents a smoking-associated mutational signature, while reduced exposure to exogenous carcinogens in HPV^+^ HNSCC favors the emergence of tumors carrying APOBEC-mediated driver mutations. Finally, Henderson et al. reported that APOBEC activity is responsible for creating driver mutations in the helical domain of *PIK3CA* gene across multiple cancers. They implicate APOBEC activity as a key driver of *PIK3CA* mutagenesis and HPV-induced transformation [41]. Moreover, three biological subtypes of HPV^+^ HNSCC could be identified basing on gene-expression data: immune related (cluster 1), epithelial–mesenchymal transition related (cluster 2), and proliferation related (cluster 3). This stratification has a prognostic relevance, with cluster 1 having the best outcome, cluster 2 the worst, and cluster 3 an intermediate survival rate [42].

## 3. The PYHIN Proteins

The five interferon-inducible proteins belonging to the PYHIN family have recently emerged as an important group of intracellular DNA sensors [5,6]. The N-terminal PYRIN domain (PYD), present in all PYHIN family members, mediates homotypic protein–protein interaction. One or more C-terminal DNA binding HIN domains are also present in every PYHIN protein, except for POP3. Of note, IFI16 has two HIN domains [7].

PYHIN proteins participate in the innate immune response as pattern-recognition receptors (PRRs), acting as whistleblowers of pathogen-associated molecular patterns (PAMPs) [5,43]. In particular, PYHIN proteins can bind virus-derived intracellular DNA through the HIN domain and are present in both the nucleus and cytosol. AIM2 and POP3 are almost exclusively cytosolic, whereas IFI16, MNDA, and PYHIN1 are mainly nuclear due to the presence of an N-terminal nuclear localization sequence (NLS) [44,45,46]. However, IFI16 can translocate to the nucleus upon pathogen DNA stimulation or acetylation [47]. PYHIN proteins, especially IFI16, are induced by type I interferon, and, conversely, they can induce type I interferon production [6].

### 3.1. Myeloid Cell Nuclear Differentiation Antigen (MNDA)

MNDA, the first human PYHIN family member to be identified, is a 55 kDa protein predominantly expressed in the nucleus of myeloid cells [48]. MNDA has been implicated in the transcriptional regulation of myeloid differentiation through interaction with the transcriptional activator–repressor YY1 [49]. Moreover, MNDA has been shown to bind nucleolin and nucleophosmin, two nucleolar proteins implicated in the maturation and biosynthesis of ribosomes [50,51]. In neutrophils, MNDA, once cleaved by pro-apoptotic caspases, localizes to the cytosol where it promotes degradation of the antiapoptotic protein myeloid cell leukemia 1 (MCL1), leading to mitochondrial dysfunction and cell death [52]. Fittingly, in septic patients, reduced cytosolic accumulation of MNDA promotes neutrophil cell survival [52].

### 3.2. Interferon-Inducible Protein 16 (IFI16)

**Figure 1:**
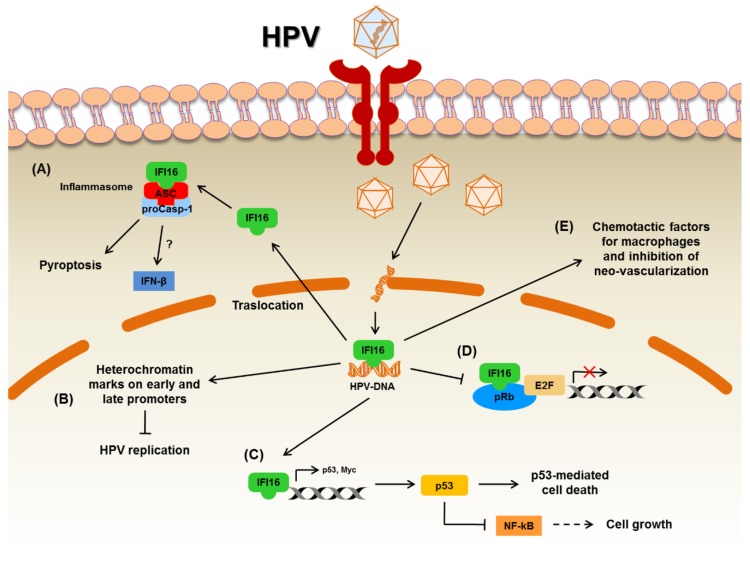
Schematic model for the putative role of IFI16 in HPV^+^ HNSCC. (**A**) IFI16 assembles with the adaptor molecule ASC and procaspase-1 (proCasp-1) to form an efficient inflammasome complex, which regulates the cleavage of caspase-1 (Casp-1) and the maturation of IL-1β. In HPV^+^ keratinocytes, IFI16 inflammasome activation triggers IFN-β release. (**B**) In HPV-infected primary human keratinocytes and human bone osteosarcoma epithelial cells, IFI16 promotes the addition of heterochromatin marks and the reduction of euchromatin marks on viral chromatin at both early and late promoters, thus impairing both viral replication and transcription. (**C**) IFI16 interacts with the promoter region of both *TP53* and *c-myc* genes, inducing their transcription. Moreover, IFI16 binds the C-terminal region of *TP53*, increasing p53-mediated transcriptional activation and cell death. p53 also blocks cell growth via suppression of NF-kB activity. (**D**) The association with pRb and the transcription factor E2F1 results in the inhibition of pRb–E2F1-mediated transcriptional repression. (**E**) IFI16 stimulates the release of chemotactic factors for macrophages and impairs neo-vascularization. Mechanisms (**A**,**B**) are more related to HPV infection response, while the others (**C**)–(**E**) are related to cancer pathogenesis.

The second identified human PYHIN family member, IFI16, can be found in three alternative spliced isoforms (i.e., A, B, and C) clustered at 85–95 kDa [43,53,54]. The predominant B isoform is present in hematopoietic, epithelial, and endothelial cells as well as fibroblasts [55,56]. Due to its role in cell cycle control and transcriptional regulation, IFI16 has been involved in DNA damage response, apoptosis, senescence, and cell growth and differentiation (Table 2) [57,58]. In particular, IFI16 can bind the C-terminal region of p53 through the amino acid sequence MFHATVAT contained in its HIN-A domain, thereby increasing p53-mediated transcriptional activation [59]. Moreover, IFI16 can also directly interact with the promoter region of both *TP53* and *c-myc* [60]. In addition, IFI16 can mediate DNA damage-induced apoptosis thanks to its ability to bind BRCA1 and activate p53-mediated cell death [61]. In contrast, its association with pRb and the transcription factor E2F1 inhibits pRb–E2F1-mediated transcriptional repression (Figure 1) [62]. Finally, IFI16 has been implicated in the pathogenesis of systemic lupus erythematosus, where it is secreted from cells as alarmin, giving rise to a common autoantigen [63,64,65,66].

The antiviral role of IFI16 has been reported for both DNA and RNA viruses. The HIN domains allow this protein to bind both double-strand (ds) and single-strand (ss) DNA in a sequence-independent manner [7]. In particular, IFI16 can act as a viral restriction factor during human cytomegalovirus (HCMV) infection by interfering with Sp1-mediated transcriptional activation of viral genes [67]. In the early phases of infection, HCMV pp65 recruits IFI16 to the major immediate-early promoter (MIEP), stimulating MIEP activity [68,69]. However, at early–late stages of infection, HCMV induces IFI16 translocation from the nucleus to the cytoplasm, thereby circumventing IFI16-mediated immune response. In particular, upon interacting with UL97, IFI16 undergoes phosphorylation, which then promotes the aforementioned nucleo-cytoplasmic shuttling. In addition to UL97, the viral tegument protein pp65 also plays a role in HCMV immune evasion by interacting with IFI16 at the level of early gene promoters such as that of the viral DNA polymerase UL54 [70].

More recently, the cellular DNA sensor, cyclic GMP-AMP synthase (cGAS), has been shown to be another important interactor of IFI16, albeit these two proteins display dissimilar functions. Specifically, upon herpes simplex virus type I (HSV-1) and HCMV infections, IFI16 interacts with cGAS through the PYRIN domain [71], but whereas IFI16 promotes an antiviral response through IFN-β induction, cGAS preferentially activates the stimulator of interferon genes (*STING*)/ TANK-binding kinase 1 (*TBK-1*)/ interferon regulatory factor 3 (*IRF3*) pathway to induce cell death [69,71]. An antiviral role of IFI16 has also been demonstrated for Kaposi’s sarcoma-associated herpes simplex virus (KHSV) and Epstein–Barr Virus (EBV) [6]. In HSV-1-infected cells, IFI16 can sense the viral genome once it has been released into the nucleus and has completely circularized. Subsequently, IFI16 translocates into the cytoplasm where it assembles with the adaptor molecule apoptosis-associated speck-like protein containing a C-terminal caspase recruitment domain (CARD) (ASC) and procaspase-1 (proCasp-1) forming the inflammasome complex, which promotes caspase-1 (Casp-1)-mediated activation of mature IL-1β. Nucleo-cytoplasmic shuttling of IFI16 translocation also triggers IRF-3-mediated IFN type I production [43]. Finally, the HSV-1 immediate–early protein ICP0 degrades IFI16 through the proteasome [72]. In response to KHSV infection, viral DNA promotes IFI16-mediated recruitment of ASC and proCasp-1 giving rise to a fully functional inflammasome in the nucleus, which upon cytoplasmic translocation drives pro-IL-1β processing [73]. Similar to what was described for KHSV, recognition of EBV DNA by IFI16 activates the ASC/Casp-1 inflammasome complex, leading to Casp-1-mediated cleavage of pro-IL-1β into its mature form. Finally, IFI16, Casp-1, and mature IL-1β can also be released from the host cells via exosomes, as a way for EBV to subvert their inflammatory functions [74].

### 3.3. Absent in Melanoma 2 Protein (AIM2)

**Figure 2:**
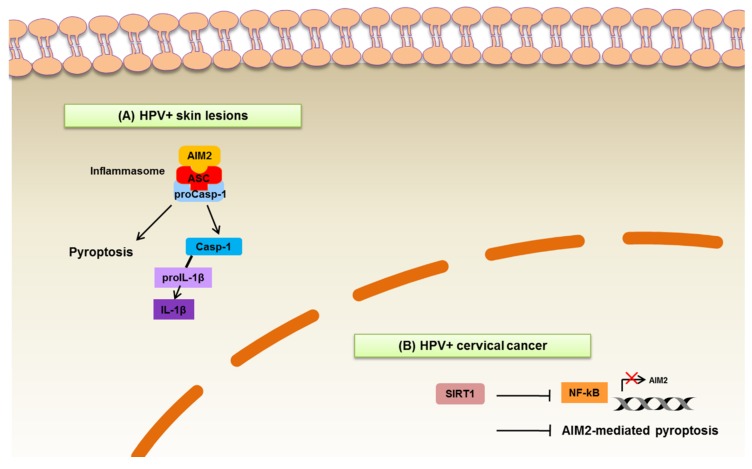
Schematic representation of the role of AIM2 in cancer and HPV infection. (**A**) In HPV^+^ keratinocytes, AIM2 binds viral DNA and interacts with the adaptor protein ASC. Conversely, ASC can interact with procaspase-1 (proCasp-1), forming an inflammasome complex. Activation of the AIM2 inflammasome results in a type of inflammatory cell death called pyroptosis. Moreover, caspase-1 (Casp-1) induces the production of IL-1β. (**B**) In HPV^+^ cervical cancer, the protein deacetylase Sirtuin 1 (SIRT1) is overexpressed and represses NF-κB-driven transcription of AIM2. Mechanism (**A**) is related to HPV infection response, while (**B**) is related to cancer pathogenesis.

Similar to IFI16, the PYHIN protein AIM2 plays an important role as a gatekeeper of host innate immunity, even though before being classified as a DNA sensor, it was initially identified among a set of genes associated with the reversal of the malignant phenotype of melanoma cells (absent in melanoma) (Figure 2) (Table 2) [75,76]. AIM2 has a HIN domain that binds directly dsDNA and a PYD domain that interacts with the PYD domain of the adaptor protein ASC. Conversely, the CARD domain of ASC can interact with the CARD domain of proCasp-1, forming an inflammasome complex [77]. Activation of the AIM2 inflammasome through phosphorylation and linear ubiquitination of ASC promotes pyroptosis [78], which is a type of cell death in part mediated by the caspase substrate gasdermin D [79]. The innate immune response is then brought to an end by autophagy, which triggers AIM2 inflammasome degradation [80]. Thus, the AIM2 inflammasome is key to the host immune response against viruses. For example, vaccinia virus, mouse cytomegalovirus (MCMV), and HPV can all be detected by AIM2 once their DNA has entered the cytoplasm [81].

Another important function of AIM2 relies on its ability to suppress neoplastic progression [82]. Indeed, in 76.9% of colorectal tumors, AIM2 displays low basal expression levels and frameshift microsatellite instability, and it is generally associated with poor prognosis [83]. AIM2 expression is also reduced in prostate cancer, whereas it is upregulated in nasopharyngeal and oral squamous cell carcinoma and lung adenocarcinoma [11]. Recent studies demonstrated that AIM2 can inhibit colorectal cancer cell proliferation leading to cell cycle arrest independent of the inflammasome [82,84]. Specifically, it appears that AIM2 can suppress the activation of DNA-PK and DNA-PK-dependent phosphorylation of AKT [85].

### 3.4. Pyrin and HIN Domain Family 1 (PYHIN1)

PYHIN1, also known as IFIX, is among the most recently identified PYHIN proteins. As a result of alternative mRNA splicing, it can be found in six isoforms (i.e., α1, α 2, β1, β2, γ1, and γ2). Being the most abundant, the isoform α1 is generally regarded as the prototype of this group. Due to the presence of an NLS, IFIX is mainly found in the nucleus [7], and like other PYHIN proteins it can be induced by interferons. Of note, IFIX is often downregulated in human breast tumors [86]. Consistent with an antitumorigenic role, IFIX acts as a negative regulator of human double minute 2 (HDM2), leading to p53 stabilization and activation of p53-mediated cell cycle arrest and apoptosis [87].

### 3.5. PYRIN Domain (PYD)-only Protein 3 (POP3)

The most recently identified member of the PYHIN family is POP3, whose genomic sequence is comprised between PYHIN1and IFI16 on the PYHIN locus. Although POP3 does not harbor a HIN domain responsible for DNA binding, it has a PYD domain, which allows it to interact with both AIM2 and IFI16, thus modulating their functions in innate immunity [44].

## 4. PYHIN Proteins in HSNCC

### 4.1. PYHIN Proteins and Human Papillomavirus Infection

In recent years, restriction factors (RFs) have emerged as key components of intrinsic immunity in response to different viruses, especially HPVs [6]. All RFs display autonomous antiviral activity in culture-based assays and can inhibit specific events occurring during viral replication. They are also constitutively expressed in a number of cell types and can be further induced upon interferon stimulation. Due to their antiviral function, they have undergone positive genetic selection during host–pathogen co-evolution, and as a result they are often antagonized by viral proteins [6]. Thus, cell lines can be generally defined as permissive or restrictive according to whether or not they allow viral replication of wild-type viruses vs. their mutant forms.

IFI16 is a sensor of exogenous DNA and acts as a viral restriction factor against HCMV, HSV-1, KHSV, EBV [6], and, more recently, HPV [88]. In a recent study performed in vitro, the role of IFI16 in controlling human papillomavirus 18 (HPV18) replication was investigated in immortalized primary human keratinocytes (NIKS) and human bone osteosarcoma epithelial cells (U2OS), both harboring HPV18 minicircles [88]. The latter provides a cellular assay system for the study of HPV genome replication, supporting the transient and stable replication of HPV. In keeping with findings from the literature, IFI16 protein silencing in NIKS cells increased viral replication [88]. Conversely, IFI16 overexpression impaired HPV18 replication in both NIKS and U2OS cell lines. In particular, IFI16 overexpression decreased viral DNA copy numbers as well as viral transcription, indicating that IFI16 is a bona fide viral restriction factor for HPV18. The mechanism of IFI16-mediated inhibition of viral replication and transcription relies on simultaneous addition of heterochromatin marks and reduction of euchromatin marks at the level of both early and late promoters of HPV18 (Figure 1, panel B). Therefore, epigenetic modification is key to the restriction activity of IFI16 against HPV in the nucleus of infected cells, a feature that does not appear to be solely restricted to herpes viruses [88].

The role of IFI16 as a restriction factor for HPV is of particular interest for HNSCC, since HPV has been detected in 22%–47% of oropharyngeal squamous cell carcinomas [2]. In this regard, recent studies have shown a protective role of IFI16 in HNSCC in vitro and in vivo as well as in HNSCC patients [8,9,10,11,12,13,14]. One of the methods recommended for prevention of HPV-related oropharyngeal cancer is vaccination. Since the majority of HPV^+^ oropharyngeal tumors are caused by HPV16 or -18, vaccination against HPV is likely to play a fundamental role in preventing HPV-related oropharyngeal cancer in the years to come [25].

Even though the role of AIM2 in HPV infection has been only marginally addressed, it is well established that the AIM2 inflammasome is crucial for the immune response to viral and bacterial infections. In particular, AIM2 is involved in DNA and RNA virus infection, such as CMV, hepatitis B virus (HBV), HPV, and influenza virus [89]. With regard to HPV, the role of the AIM2 inflammasome has been investigated in keratinocytes infected with HPV16. In these cells, binding of viral DNA triggers AIM2 inflammasome activation and release of IL-1β and IL-18 (Figure 2, panel A), whereas IFI16 activation leads to IFN-β secretion (Figure 1, panel A). Interestingly, AIM2-silenced cells showed increased IFN-β secretion, while inhibition of IFI16 upregulated IL-1β production, indicating the existence of a crosstalk between these two viral sensors upon HPV infection [90]. In this regard, the authors suggest that IFI16 may act as a negative regulator of AIM2 inflammasome activation in keratinocytes.

Finally, an interesting interplay between AIM2 and the NAD^+^-dependent protein deacetylase Sirtuin 1 (SIRT1) has recently emerged in HPV-induced cervical cancer. In these tumor cells, aberrant expression of SIRT1 inhibits NF-κB-mediated transcription of AIM2 through destabilization of v-rel reticuloendotheliosis viral oncogene homolog B (RELB) mRNA. Moreover, knockdown of SIRT1 not only result in pyroptosis of HPV-infected cervical cancer cells, such as SiHa cells, but it also induces pyroptotic cell death of naïve cervical cancer cells by releasing extracellular vesicles carrying AIM2 inflammasome proteins (Figure 2, panel B) [91]. Fittingly, SIRT1 expression correlates with poor survival in cervical cancer patients [91]. Thus, it will be extremely interesting to verify in future studies whether similar mechanisms hold true for HNSCC as well.

### 4.2. The Role of IFI16 and AIM2 Proteins in HSNCC

Over the last two decades, the role of several PYHIN proteins, especially IFI16 and AIM2, in HNSCC has been widely investigated (Table 3).

In 2004, Azzimonti et al. analyzed 36 HNSCCs—27 tumors were from the larynx, whereas nine originated from the oropharynx—and found that 17 out of the 19 tumors positive for HPV had high levels of IFI16 protein expression, as judged by immunohistochemical score [8]. Moreover, IFI16 protein expression in the 36 HNSCCs was inversely correlated with the proliferation index—i.e., high Ki67 expression levels were found in low IFI16 expressors. Furthermore, low-proliferating IFI16^+^ tumors displayed marked expression of pRb, which was consistent with increased overall survival for patients with higher IFI16 expression. Unfortunately, the absence of stratification for HPV prevalence in this study does not allow generalizing their findings relative to patient survival, a limitation that is supported by the fact that patients with HPV^−^ and HPV^+^ tumors display similar overall survival rates. Nevertheless, this study contains a series of important preliminary results that warrant further investigation. In particular, it will be crucial to determine whether the higher IFI16 immunostaining of low-grade, less-aggressive tumors is indeed related to the antiproliferative activity of PYHIN proteins.

By the means of in vitro studies, it was observed that ectopic expression of IFI16 in an IFI16-null HNSCC-derived cell line (HNO136) suppressed both proliferation and transforming activity of these cells in vitro, indicating that IFI16 exerts a potent antiproliferative effect in HNSCC [9]. Congruously, IFI16 overexpression in the IFI16^+^ HNSCC-derived cell line HNO124, harboring wild-type p53, correlated with low growth rates and poor colony formation activity. By contrast, IFI16 overexpression in another IFI16^+^ HNSCC-derived cell line (HNO150), harboring a mutated p53, failed to exert antiproliferative activity, suggesting that IFI16-mediated inhibition of cell growth probably depends on the presence of a functional p53 protein. In the same study, the authors observed that IFI16 restoration in HNO136 increased doxorubicin-induced cell death through G_2_/M phase arrest. Specifically, fluorescence-activated cell sorter (FACS) analysis revealed that ectopic expression of IFI16 in HNO136 cells caused a more pronounced G_2_ arrest in comparison with their non-transduced counterparts. The lack of caspase-3 activation and p53 transcriptional activity in these cells ruled out the possibility that doxorubicin-treated cells were dying from apoptosis.

The antiproliferative role of IFI16 was subsequently confirmed in vivo using a tumorigenicity assay [10]. Upon retroviral restoration of IFI16 expression, HNO136 cells were subcutaneously inoculated in nude mice, and their growth properties were compared to those of non-transduced HNO136 cells. The xenografts performed with IFI16-transduced HNO136 cells produced significantly smaller tumors with a higher volume of necrosis and percentage of apoptotic cells. Necrotic changes positively correlated with anti-angiogenic role of IFI16 demonstrated by analyzing the tumor microvessel density. Since IFI16 expression correlated with infiltration of CD45^+^ cells at the stromal–epithelial junction, the authors performed chemotaxis experiments in vitro to determine whether IFI16 was directly involved in blood cell recruitment. The supernatants from IFI16 transduced HNO136 cells showed chemoattractive activity on mouse macrophages. This result was confirmed by the detection of CD68^+^ or CD14^+^ macrophage infiltration in the xenografts by immunohistochemistry. Overall, these data indicate that IFI16 exerts an anti-tumoral activity in vivo by stimulating apoptosis of tumor cells, impairing neo-vascularization, and releasing chemotactic factors, which in turn recruit macrophages (Figure 1, panel E).

*IFI16* and *AIM2* genes are overexpressed in oral squamous cell carcinomas [11]. Knockdown of IFI16 or AIM2 in oral cancer cell lines abolished cell growth as well as apoptosis, followed by a decrease in NF-κB activity. The introduction of wild-type p53 in these cell lines blocked cell growth and induced apoptosis via suppression of NF-κB activity (Figure 1, panel C). Finally, co-expression of IFI16 and AIM2 significantly enhanced cell growth in p53 null cells. Conversely, the expression of IFI16 and/or AIM2 in cells bearing wild-type p53 repressed cell growth. Furthermore, IFI16 and AIM2 acted in concert to increase NF-κB signaling in p53 null cells. Thus, altogether these data strongly favor a model whereby the expression of IFI16 and AIM2 plays a pro-oncogenic role in cells lacking a functional p53. However, the fact that oral carcinomas are rarely related to HPV infection can lead us to speculate that these results may only apply to HPV^−^ HNSCCs.

Positivity for p16 and IFI16 was observed by immunohistochemistry in 63.6% and 45.5% of oropharyngeal squamous cell carcinomas, respectively [12]. In keeping with the literature, patients with p16^+^ tumors had a better survival compared to that of their p16 negative counterparts. On the other hand, the expression of IFI16 was not directly related to p16, and there were no significant differences in survival rates between IFI16 positive and negative patients. The absence of correlations may be related to the small sample size of the study or to the fact that p16 may not be a reliable marker for HPV infection, as put forward previously [27,28].

In 2014, Mazibrada et al. analyzed IFI16 expression by immunohistochemistry in a larger sample—i.e., 224 specimens of head and neck precancerous and malignant lesions [13]. The strength of this study was the evaluation of HPV infection not just with p16 immunohistochemistry but also with two different PCR-based assays. The authors evaluated the expression of HER-2/neu, pStat3, Sox2, IFI16, and Ki67. They found HPV DNA in 24% of cases with a predominant HPV16 genotype. HPV^+^ samples had higher HER-2/neu, pStat3, and IFI16 expression compared to the HPV^−^ lesions. An inverse correlation between IFI16 expression and Sox2/Ki67 activity was also observed. Moreover, there was a positive correlation between pStat3 and IFI16 in HPV^+^ lesions. The authors thus concluded that HPV could trigger HER2/neu, pStat3, and IFI16 expression and that high levels of pStat3 and IFI16 were suggestive of a synergistic pro-apoptotic action of these two proteins in HPV^+^ lesions. These findings are in good agreement with the observation that IFI16 is a pStat3 target and that pStat3 cooperates with IFI16 in inducing medullary thyroid carcinoma growth arrest [92].

Based on the aforementioned data, Riva et al. have more recently shown a protective role of IFI16 in HNSCC patients [14]. Specifically, the authors analyzed the expression of IFI16 and AIM2 in 34 patients undergoing surgical treatment for HNSCC and their possible correlation with the HPV infection status, clinical characteristics, and survival. Besides the PYHIN genes IFI16 and AIM2, the authors also evaluated *TP53*, *NOTCH1*, *MET*, *PD-L1*, and *APOBEC3* family members. Twenty-four patients were HPV^−^, whereas 10 were HPV^+^. While HPV^−^ HNSCCs showed reduced levels of *IFI16, APOBEC3A* and *APOBEC3B* gene expression, all three genes were upregulated in HPV^+^ tumors. Interestingly, *AIM2* gene expression was mainly unmodified in HPV^+^ HNSCCs in comparison with HPV^−^ tumors, where AIM2 was mostly found to be overexpressed (10% vs. 50%). In HPV^+^ tumors, *IFI16* expression positively correlated with *APOBEC3A* expression. Furthermore, the upregulation of IFI16 positively associated with a lower incidence of nodal metastases in HPV^−^ HNSCCs. In contrast, the HPV^+^ group did not show any statistically significant correlation with clinical characteristics. This study was the first demonstration of a positive role of IFI16 in modulating a clinical feature in HPV^−^ tumors. Lastly, IFI16 or AIM2 low expressors displayed a worse prognosis, especially with regard to overall survival. However, the correlation between nodal metastases and IFI16 may be a confounding factor due to the better prognosis of patients without nodal metastases. 

## 5. Conclusions and Future Perspectives

In conclusion, from the literature reviewed above, it appears that the different molecular profiles and prognoses of HPV^−^ vs. HPV^+^ HNSCCs may be partly ascribed to the emerging etiopathological role played by PYHIN proteins in these tumors. In this regard, particular attention has been paid to the functions of the two PYHIN family members IFI16 and AIM2. On the one hand, IFI16 appears to be mainly overexpressed in HPV^+^ tumors compared to their HPV^−^ counterparts, which is consistent with its well-established role of viral restriction factor. On the other hand, AIM2 seems to be predominantly overexpressed in HPV^−^ HNSCC. It is however important to point out that in 50% of HPV^+^ tumors AIM2 expression levels are unchanged, supporting the hypothesis that this intracellular sensor of viral DNA might be differentially regulated in HPV^+^ vs. HPV^−^ HNSCC, an exciting possibility that should be further explored.

Interestingly, IFI16 is inversely associated with nodal metastases in HPV^−^ HNSCCs, suggesting a protective role against the lymphatic diffusion of tumor cells also in the absence of HPV infection. This is probably due to the ability of IFI16 to orchestrate multiple transcription factors, as previously demonstrated during B-cell differentiation [93]. Further studies with larger samples are clearly needed to clarify this important issue.

Overall, patients with low levels of IFI16 and AIM2 expression seem to have a worse prognosis. However, the correlation between nodal metastases and IFI16 may represent a confounding factor in such survival analyses. Thus, the prognostic value of PYHIN proteins in human HNSCC awaits additional studies on larger patient cohorts. Such studies should allow the identification of new druggable targets and provide the rationale for de-intensification trials on HPV^+^ patients.

## Figures and Tables

**Table 1:** Molecular profiles of human papillomavirus (HPV)^−^ and HPV^+^ head and neck squamous cell carcinoma (HNSCC).

**HPV^−^ HNSCC**	Losses of chromosomes 3p and 9p *TP53* mutations Loss of the tumor suppressor gene *CDKN2A* *PIK3CA* amplification and/or mutation in 34% of cases Overexpression of *EGFR and MET* Smoking-associated mutational signature
**HPV^+^ HNSCC**	Lower average number of mutations per tumor Wild-type *TP53* Rare loss of the tumor suppressor gene *CDKN2A* *PIK3CA* amplification and/or mutation in 56% of cases APOBEC-mediated driver mutations

**Table 2:** Interferon-inducible protein 16 (IFI16) and absent in myeloma 2 protein (AIM2) proteins: general features.

	Intracellular localization	Functions	Interaction with Other Proteins
**IFI16**	us aNuclend cytoplasm	- DNA damage response, apoptosis, senescence, and cell growth and differentiation - Activation of an efficient inflammasome complex - Binding double strand (ds) and single strand (ss) DNA in a sequence-independent manner - Viral restriction factor as DNA intracellular sensor	- Increase of p53-mediated transcriptional activation - Interaction with the promoter region of p53 and c-myc - Binding BRCA1 and activation of p53-mediated cell death - Inhibition of pRb–E2F1-mediated transcriptional repression - Binding the adaptor protein ASC to form an inflammasome
**AIM2**	Cytoplasm	- Activation of an efficient inflammasome complex - Viral restriction factor as DNA intracellular sensor - Suppression of the activation of DNA-PK and DNA-PK-dependent phosphorylation of AKT	- Binding the adaptor protein ASC to form an inflammasome

**Table 3:** The role of IFI16 and AIM2 proteins in HNSCC: a review of the literature.

Reference	Type of Study	Main Results	Strengths	Limits
Azzimonti et al., 2004 [8]	IHC ^1^ on 36 HNSCC specimens.	- Higher IFI16 expression in HPV^+^ HNSCC; - Inverse correlation between IFI16 and Ki67 expression; - Better prognosis for patients with high IFI16.	- First study correlating IFI16 expression with HPV infection in HNSCC; - Evidence of IFI16 antiproliferative activity.	- No stratification for HPV infection in survival analysis; - No tumors from oral cavity.
De Andrea et al., 2007 [9]	In vitro (HNSCC-derived cell lines).	- IFI16 restoration inhibits both cell growth and transforming activity in vitro; - IFI16-mediated inhibition of cell growth depends on the presence of a functional p53; - IFI16 increases doxorubicin-induced cell death by G_2_/M phase arrest.	- In vitro demonstration of the antiproliferative activity of IFI16 in a p53-dependent fashion.	- No analysis of IFI16 activity in vivo.
Mazibrada et al., 2010 [10]	In vivo tumorigenicity assay (nude mice xenografts).	- IFI16 exerts an anti-tumoral activity in vivo by promoting apoptosis of tumor cells, inhibiting neo-vascularization, by increasing the recruitment of macrophages through the release of chemotactic factors.	- In vivo demonstration of the functional role of IFI16in HNSCC.	- Evaluation only limited to some HNSCC-derived cell lines.
Kondo et al., 2012 [11]	Gene expression profiling on 28 HNSCC specimens.	- Over-expression of *IFI16* and *AIM2* genes in oral cancer; - Knockdown of *IFI16* or *AIM2* in cell lines from oral cancer suppresses cell growth and apoptosis, accompanied by the downregulation of NF-κB activity; - In p53-deficient cells, the expression of IFI16 and AIM2 may have transformation potential.	- Evaluation of NF-κB signaling; - First evaluation of AIM2 in HNSCC.	- No assessment of HPV infection status; - No tumors from pharynx and larynx.
Yamauchi et al., 2013 [12]	IHC on 22 HNSCC specimens.	- The expression of IFI16 is not associated with p16; - IFI16 positive and negative patients have similar survival rates.	- Survival analysis based on IFI16	- p16 is not the perfect marker for HPV infection; - Small sample; - No stratification for HPV infection in survival analysis.
Mazibrada et al., 2014 [13]	IHC on 224 head and neck precancerous and malignant lesions.	- Higher expression of HER-2/neu, pStat3, and IFI16 expression in HPV^+^ lesions; - Inverse correlation between IFI16 expression and Sox2/Ki67 activity; - Positive correlation between pStat3 and IFI16 expression in HPV^+^ lesions.	- Large sample - Evaluation of HPV infection with not only p16 IHC but also two different PCR-based assays - Observation of pStat3 and IFI16 synergistic pro-apoptotic effects in HPV^+^ lesions.	- No survival analysis.
Riva et al., 2019 [14]	mRNA expression levels in 34 specimens of HNSCC	- Upregulation of IFI16, APOBEC3A, and APOBEC3B in HPV^+^ HNSCCs; - *AIM2* gene expression levels are predominantly unchanged in HPV^+^ HNSCCs compared to their HPV^−^ counterparts, in which AIM2 is predominantly upregulated; - Positive correlation between IFI16 and APOBEC3A expression in HPV^+^ HNSCCs; - Upregulation of IFI16 correlates with lower occurrence of nodal metastases in HPV^−^ HNSCCs; - Worse prognosis for patients with downregulated IFI16 or AIM2.	- Demonstration of protective role of IFI16 in both HPV^+^ and HPV^−^ HNSCCs.	- Small HPV^+^ sample group (10 patients) - Short follow-up (mean follow-up: 19 months).

^1^ IHC = immunohistochemistry.
